# Extracorporeal Shockwave Therapy Versus Ultrasound Therapy for Plantar Fasciitis: A Systematic Review and Meta-Analysis

**DOI:** 10.7759/cureus.20871

**Published:** 2022-01-02

**Authors:** Zeyana Al-Siyabi, Mohammad Karam, Ethar Al-Hajri, Abdulmalik Alsaif, Mohammad Alazemi, Ahmed A Aldubaikhi

**Affiliations:** 1 Podiatry, Starcare Hospital, Muscat, OMN; 2 Podiatry, University of Huddersfield, Huddersfield, GBR; 3 School of Medicine, University of Leeds, Leeds, GBR; 4 Medicine, Farwaniya Hospital, Ministry of Health, Kuwait City, KWT; 5 Medicine, Walsall Healthcare NHS Trust, West Midlands, GBR; 6 Medicine, King Abdullah International Medical Research Center, Riyadh, SAU; 7 College of Medicine, King Saud Bin Abdulaziz University for Health Sciences, Riyadh, SAU

**Keywords:** functional impairment, heel pain, ultrasound therapy, extracorporeal shockwave therapy, plantar fasciitis

## Abstract

The objective of this study was to compare the outcomes of extracorporeal shockwave therapy (ESWT) versus ultrasound therapy (UST) in plantar fasciitis. A systematic review and meta-analysis were performed. An electronic search identifying studies comparing ESWT and UST for plantar fasciitis was conducted. Primary outcomes were morning and activity pain, functional impairment, and the American Orthopaedic Foot and Ankle Society (AOFAS) scale score. Secondary outcomes included the fascial thickness, primary efficacy success rate, activity limitations, pain intensity, and satisfaction. Seven studies enrolling 369 patients were identified. No significant difference was found between ESWT and UST for functional impairment (mean difference [MD] = −2.90, P = 0.22), AOFAS scale score (MD = 35, P = 0.20), and pain in the first steps in the morning (MD = −4.72, P = 0.39). However, there was a significant improvement in pain during activity for the ESWT group (MD = −1.36, P = 0.005). For secondary outcomes, ESWT had improved results in terms of primary efficacy success rate, activity limitations, and patient satisfaction. The reduction of plantar fascia thickness showed no significant difference. Pain intensity after treatment had varied results amongst included studies. In conclusion, ESWT is superior to UST for plantar fasciitis as it improves pain activity and intensity, primary efficacy success rate, and activity limitations.

## Introduction and background

Plantar fasciopathy or plantar fasciitis is one of the most common foot disorders and occurs in approximately 10% of the population throughout their life [1]. Although it was defined as an inflammatory syndrome, recent studies have emphasised that plantar fasciopathy is more likely to be a degenerative process associated with multifactorial aetiology [2,3]. Factors are thought to be anatomical (such as pes planus and pes cavus) or biomechanical (such as excessive external rotation and subtalar joint overpronation) or environmental (such as obesity and inappropriate footwear) [4-7]. Plantar fasciitis is diagnosed clinically and MRI imaging is a second-line diagnostic test to confirm the diagnosis and rule out other foot disorders due to its considerable costs [8,9]. Typical presentations are throbbing, burning, or piercing medial heel pain, especially in the first steps in the morning or after a prolonged rest period [3]. The pain typically decreases after a few steps but may return with continued weight-bearing activities [3]. If untreated, pain may last for months or years [3]. Conservative treatments, such as activity modification, oral analgesics, ice massage, stretching exercises, orthotics, and corticosteroid injections, can help with disabling pain. Patients with chronic plantar fasciitis can consider other treatment options, including extracorporeal shockwave therapy (ESWT), ultrasound therapy (UST), low-level laser therapy (LLLT), or surgical plantar fasciotomy [10,11].

ESWT comprises focused pulses of high-pressure sound waves to bombard damaged tissues aiming to minimise pain and symptoms associated with plantar fasciitis. They were initially used for medical purposes in the management of renal calculi by lithotripsy. Subsequently, shockwaves were utilised in the treatment of ununited fractures [12,13]. Years later, they became popular in Germany for certain musculoskeletal complaints, including calcifying tendonitis epicondylitis and plantar fasciitis [14]. ESWT has been used as an alternative to surgery for patients with long-term, recalcitrant plantar heel pain. The mechanism of the action of shock waves on soft-tissue conditions remains speculative. Experts propose the pulses bombard the central nervous system by causing alterations in the permeability of cell membranes inhibiting the transmission of painful stimuli resulting in pain relief, while others contend that they stimulate the healing cascades by essentially re-injuring the tissues [15,16].

Therapeutic ultrasound (US) is one of the physiotherapeutic modalities commonly used in the management of soft tissue disorders such as plantar fasciitis. US is a high-frequency wave that produces thermal or non-thermal effects depending on the frequency, intensity, duration of pulses, and injury type [17]. It was reported that US has advantages in the healing of soft tissue [18,19]. It has a base unit for generating electrical signals that transmit through biological tissues causing a rise in tissue temperature and metabolism and thus enhancing blood circulation [20]. Ultrasonic energy has also been purported to affect the chemical activity of tissues by increasing the permeability of the cell membranes and regulating the molecular structures and protein production, all possibly resulting in the promotion of tissue recovery and a shorter healing process [19]. Nonetheless, there is a lack of high-quality scientific evidence to support the practical use of UST in the management of musculoskeletal conditions.

There are currently no systematic reviews or meta-analyses that solely compare the use of ESWT against UST for plantar fasciitis treatment although they have been reported in several recent randomised controlled trials (RCTs) as well as non-randomised cohort studies [21-27]. Therefore, it is imperative to conduct the first review within the literature regarding this topic.

This article was previously posted to the medRxiv preprint server on September 22, 2020 (https://www.medrxiv.org/content/10.1101/2020.09.20.20198168v1).

## Review

Methods

A systematic review and meta-analysis were conducted as per the Preferred Reporting Items for Systematic Reviews and Meta-Analyses (PRISMA) guidelines [28].

Eligibility Criteria

All RCTs and observational studies comparing ESWT with UST for plantar fasciitis treatment were included. ESWT was the intervention group of interest and UST was the comparator. All patients were included irrespective of age, gender, or co-morbidity if they belonged to either a study or control group. Case reports and cohort studies where no comparison was conducted were excluded from the review process.

Primary Outcomes

The primary outcomes are pain in the morning and during activity, functional impairment, and the American Orthopaedic Foot and Ankle Society (AOFAS) scale score. The pain was reported using the visual analogue scale (VAS) during the morning pain when taking the first steps and during activities like exercise or walking [23,24,26]. Using a self-administered questionnaire (University of Peloponnese Pain, Functionality, and Quality of Life Questionnaire), functional impairment was evaluated on a five-point Likert scale, before treatment, immediately after, and at four-week follow-up [27]. The AOFAS scale was used to measure foot functionality and range of motion [24].

Secondary Outcomes

The secondary outcomes included are fascial thickness before and after treatment, primary efficacy success rate, activity limitations, pain intensity, and patient satisfaction.

Literature Search Strategy

Two authors independently searched the following electronic databases: MEDLINE, EMCare, Embase, CINAHL, and the Cochrane Central Register of Controlled Trials (CENTRAL). The last search was run on 1 December 2021. Thesaurus headings, search operators, and limits in each of the above databases were adapted accordingly. In addition, the World Health Organization International Clinical Trials Registry (http://apps.who.int/trialsearch/), ClinicalTrials.gov (http://clinical-trials.gov/), and ISRCTN register (http://www.isrctn.com/) were searched for details of ongoing and unpublished studies. No language restrictions were applied in our search strategies. The search terminologies included “Extracorporeal Shockwave Therapy”, “Ultrasound Therapy”, “Plantar Fasciitis” and “Plantar Fasciosis”. All terms were combined with adjuncts of “and” as well as “or”. The bibliographic lists of relevant articles were also reviewed.

Selection of Studies

The title and abstract of articles identified from the literature searches were assessed independently by two authors. The full texts of relevant reports were retrieved and those articles that met the eligibility criteria of our review were selected. Any discrepancies in study selection were resolved by discussion between the authors.

Data Extraction and Management

An electronic data extraction spreadsheet was created in line with Cochrane’s data collection form for intervention reviews. The spreadsheet was pilot-tested in randomly selected articles and adjusted accordingly. Our data extraction spreadsheet included study-related data (first author, year of publication, country of origin of the corresponding author, journal in which the study was published, study design, study size, clinical condition of the study participants, type of intervention, and comparison), baseline demographics of the included populations (age and gender), and primary and secondary outcome data. Two authors cooperatively collected and recorded the results and any disagreements were solved via discussion.

Data Synthesis

Data synthesis was conducted using the Review Manager 5.3 software (The Cochrane Collaboration, London, UK). The extracted data were entered into Review Manager by two independent authors. The analysis used was based on fixed and random effects modelling. The results were reported in forest plots with 95% confidence intervals (CIs).

For continuous outcomes, the mean difference (MD) was calculated between the two groups. A positive MD for pain, functional impairment, and AOFAS scale score would favour the ESWT group, a negative MD would favour the US therapy group, and an MD of 0 would favour neither group.

Assessment of Heterogeneity

Heterogeneity among the studies was assessed using the Cochran Q test (χ2). Inconsistency was quantified by calculating I2 and interpreted using the following guide: 0% to 25% may represent low heterogeneity, 25% to 75% may represent moderate heterogeneity, and 75% to 100% may represent high heterogeneity.

Results

Literature Search Results

**Figure 1:**
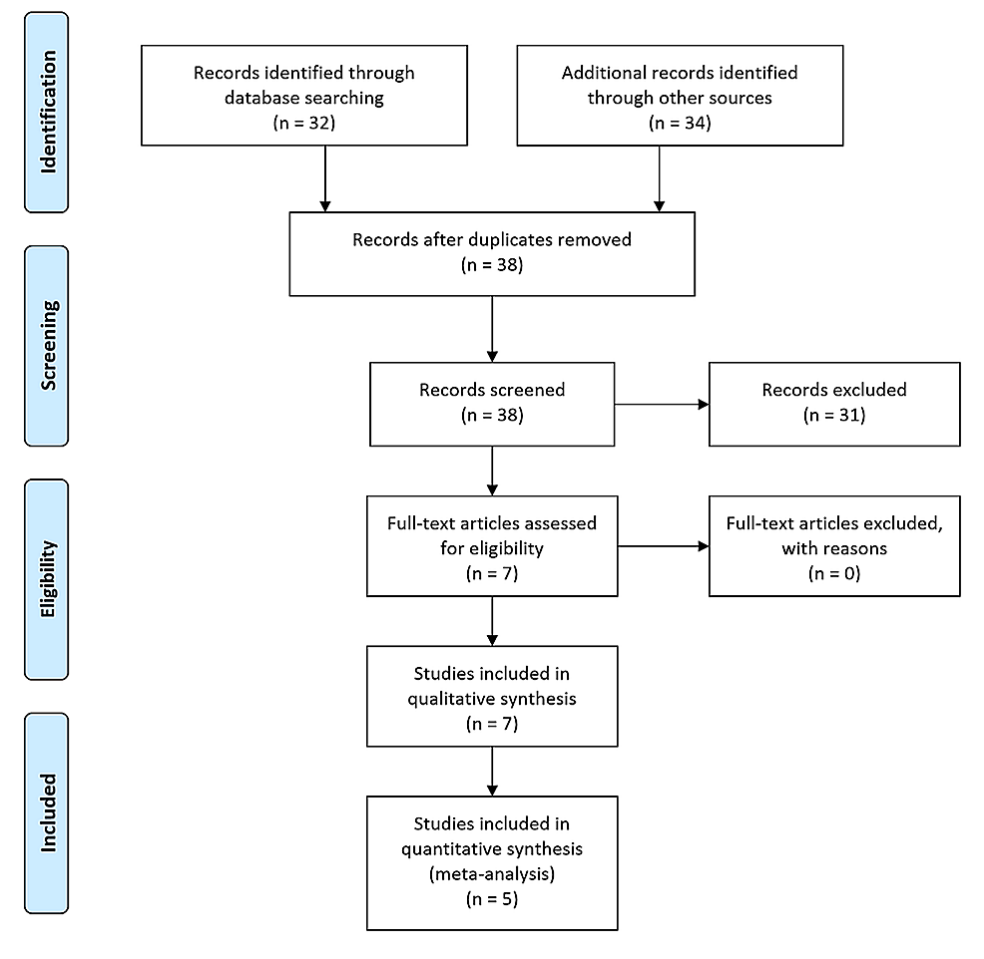
PRISMA flow diagram. The PRISMA diagram details the search and selection processes applied during the overview. PRISMA, Preferred Reporting Items for Systematic Reviews and Meta-Analyses.

The search strategy retrieved 63 studies. After a thorough screening of the retrieved articles, seven studies in total were identified, which met the eligibility criteria (Figure 1).

Description of Studies

Baseline characteristics of the included studies are summarised in Table 1.

**Table 1:** Baseline characteristics of the included studies. ESWT, extracorporeal shockwave therapy; UST, ultrasound therapy; LLLT, low-level laser therapy; rESWT, radial extracorporeal shockwave therapy; RCT, randomised controlled trial

Study	Year	Country	Journal	Study design	Total study sample	Interventions compared
Cheing et al. [21]	2007	China	Shock Waves	Cohort study	37	ESWT vs. UST vs. no treatment
Greve et al. [22]	2009	Brazil	Clinics	RCT	32	rESWT vs. UST
Konjen et al. [23]	2015	Thailand	Journal of the Medical Association of Thailand	RCT	30	rESWT vs. UST
Ulusoy et al. [24]	2017	Turkey	The Journal of Foot & Ankle Surgery	RCT	60	ESWT vs. UST vs. LLLT
Akınoğlu et al. [25]	2017	Turkey	Pain Medicine	RCT	54	rESWT + exercise vs. UST + exercise vs. exercise
Akınoğlu and Köse [26]	2018	Turkey	Journal of Exercise Rehabilitation	RCT	54	rESWT + exercise vs. UST + exercise vs. exercise
Dedes et al. [27]	2019	Greece	Acta Informatica Medica	Cohort study	159	rESWT vs. UST vs. conventional therapy

Cheing et al. [21] conducted a dual-centre prospective cohort study that included 37 participants with chronic plantar fasciitis. Participants were allocated into one of three groups, which received ESWT, UST, or no treatment (control). Only one of the two participating clinics was equipped with the ESWT machine and hence patients attending this clinic were allocated to the ESWT group (12 patients). Patients in the second clinic were randomly assigned to either the US (15 patients) or the control group (10 patients) by drawing lots.

Greve et al. [22] conducted a single-centre randomised, prospective, and comparative clinical study that included 32 patients with chronic plantar fasciitis. Participants were divided into two groups in accordance with randomly drawn numbers: 16 in the radial ESWT (rESWT) group and 16 in the UST group.

Konjen et al. [23] conducted a single-centre prospective randomised clinical trial that included 30 patients with chronic plantar fasciitis. A computerised random number generator was used to conduct block randomisation into two groups: 15 patients in the rESWT group and 15 patients in the UST group.

Ulusoy et al. [24] conducted a single-centre prospective randomised clinical trial that included 60 patients with chronic recalcitrant plantar fasciitis. Using the stratified block randomization method according to gender and body mass index, participants were randomised into three treatment groups: 20 patients in the ESWT group, 20 in the UST group, and 20 in the LLLT group.

Akınoğlu et al. (2017) [25] conducted a single-centre prospective randomised controlled trial that included 54 patients with chronic plantar fasciitis attending physical medicine and rehabilitation clinic. The sealed envelope method was used for randomisation of the study sample into three groups: 24 patients in the rESWT and exercise group, 26 patients in the UST and exercise group, and 28 patients in the exercise group.

Akınoğlu and Köse (2018) [26] conducted a single-centre prospective randomised controlled trial that included 54 patients with chronic plantar fasciitis attending physical medicine and rehabilitation clinic. This is the same study group as Akınoğlu et al. (2017) [25]; however, the more recent study [26] reported different outcomes. The sealed envelope method was used for randomisation of the study sample into three groups: 24 patients in the rESWT and exercise group, 26 patients in the UST and exercise group, and 28 patients in the exercise group.

Dedes et al. [27] performed a single-centre prospective cohort study that included 156 patients with chronic plantar fasciitis attending an orthopaedic clinic. The study period was from February 2015 to August 2017. The study groups included 88 patients for the rESWT group, 56 patients for the UST group, and 15 patients for the control group.

Primary Outcomes

**Figure 2:**
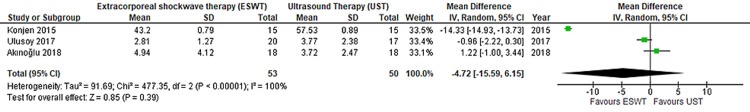
Forest plot of ESWT vs. UST – morning pain. Quantitative analysis showing the mean difference in pain in the morning reported by Konjen et al. (2015) [23], Ulusoy et al. (2017) [24], and Akınoğlu and Köse (2018) [26]. ESWT, extracorporeal shockwave therapy; UST, ultrasound therapy.

Morning and activity pain: In Figure 2, morning pain was reported in Konjen et al. [23], Ulusoy et al. [24], and Akınoğlu and Köse (2018) [26] enrolling 73 patients. There was no statistically significant difference seen in the MD analyses showing a lower level of pain in the morning for the ESWT group (MD = −4.72, CI = −15.59 to 6.15, P = 0.39). A high level of heterogeneity was found amongst the studies (I2 = 100%, P < 0.00001).

**Figure 3:**
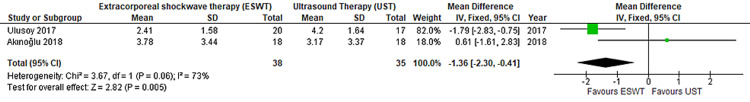
Forest plot of ESWT vs. UST – activity pain. Quantitative analysis showing the mean difference in pain during activity reported by Ulusoy et al. (2017) [24] and Akınoğlu and Köse (2018) [26]. ESWT, extracorporeal shockwave therapy; UST, ultrasound therapy.

In Figure 3, activity pain was reported in Ulusoy et al. [24] and Akınoğlu and Köse (2018) [26] enrolled 73 patients. There was a statistically significant difference seen in the MD analyses showing a lower level of pain during activity for the ESWT group (MD = −1.36, CI = −2.30 to −0.41, P = 0.005). A medium level of heterogeneity was found amongst the studies (I2 = 73%, P = 0.06).

**Figure 4:**
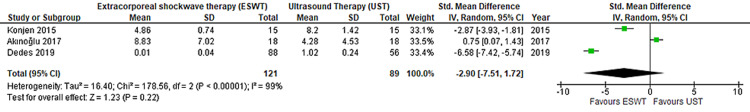
Forest plot of ESWT vs. UST – functional impairment. Quantitative analysis showing the standard mean difference in functional impairment reported by Konjen et al. (2015) [23], Akınoğlu et al. (2017) [25], and Dedes et al. (2019) [27]. ESWT, extracorporeal shockwave therapy; UST, ultrasound therapy.

Functional impairment: In Figure 4, functional impairment was reported by Konjen et al. [23], Akınoğlu et al. (2017) [25], and Dedes et al. [27] enrolling 106 patients. There was no significant statistical difference seen in the standard MD analyses showing a higher functional impairment in the ESWT group compared to the UST group (standard MD = −2.90, CI = −7.51 to 1.72, P = 0.22). A high level of heterogeneity was found amongst the studies (I2 = 99%, P <0.00001).

**Figure 5:**
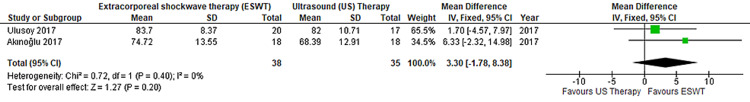
Forest plot of ESWT vs. UST – AOFAS scale score. Quantitative analysis showing the standard mean difference in UST reported by Ulusoy et al. (2017) [24] and Akınoğlu et al. (2017) [25]. ESWT, extracorporeal shockwave therapy; UST, ultrasound therapy.

AOFAS scale score: In Figure 5, the AOFAS scale score was reported by Ulusoy et al. [24] and Akınoğlu et al. (2017) [25] enrolling 38 patients. There was no statistically significant difference seen in the standard MD analyses, showing a higher AOFAS scale score for the ESWT group (MD = 35, CI = −1.78 to 8.38, P = 0.20). A low level of heterogeneity was found amongst the studies (I2 = 0%, P = 0.40).

Secondary Outcomes

Fascial thickness: According to Ulusoy et al. [24], the fascial thickness was measured on MRI from coronal and sagittal planes. There was a significant decrease in the thickness of the fascia in both groups after treatment (P < 0.001), but no statistically significant difference was found between the two groups in the reduction of thickness [24].

Primary efficacy success rate: Ulusoy et al. [24] used the reduction of heel pain as a measurement of primary efficacy rate, which was detected in 65% of the ESWT group and 23.5% of the UST group. In the comparison, the ESWT group was found to be more effective than the UST group, with a significant difference found between the two groups (P = 0.012) in the success rate [24].

Activity limitations: Activity limitations were assessed in three studies using different measurements [24,25,27]. Based on Ulusoy et al. [24] and Dedes et al. [27], there was a reduction in the activity limitations in both groups, but in comparison, ESWT treatment modality was more effective than UST (P < 0.05). However, Akınoğlu et al. [25] showed that the reduction in the activity limitations was most marked in the UST group as compared to the ESWT group (P < 0.05).

Pain intensity: Pain intensity after treatment was reported to be significantly (P < 0.05) lower for the UST group than the ESWT group in Akınoğlu et al. [25] and Akınoğlu and Köse [26]. Conversely, Dedes et al. [27] reported significantly improved results in the ESWT group both immediately after the treatment and after a four-week follow-up (P < 0.001). Both Cheing et al. [21] and Greve et al. [22] concluded that ESWT is potentially more effective in reducing pain intensity with no significant difference between the two groups.

Treatment satisfaction: Konjen et al. [23] reported patient satisfaction to be higher in the rESWT group than the UST group, with 80% and 33% of patients, respectively, rating their treatment satisfaction as “very satisfied”.

Methodological Quality and Risk of Bias Assessment

The Cochrane Collaboration’s tool was used to assess the quality of the RCTs included in the study (Table 2). For non-randomised studies, the Newcastle-Ottawa scale [29], which offers a star system for analysis, was used to assess the quality of the studies (Table 3). Although Cheing et al. [21] and Dedes et al. [27] showed low comparability, the study had a high quality for selection and exposure.

**Table 2:** Bias analysis of the randomised trials using the Cochrane Collaboration’s tool. Domain 1 = random sequence generation (selection bias); Domain 2 = allocation concealment (selection bias); Domain 3 = blinding of participants and personnel (performance bias); Domain 4 = blinding of outcome assessment (detection bias); Domain 5 = incomplete outcome data (attrition bias); Domain 6 = selective reporting (reporting bias); Domain 7 = other bias.

Authors	Bias	Authors’ judgement	Support for judgement
Greve et al. [22]	Domain 1	Low risk	A list of random numbers used
	Domain 2	High risk	Allocated based on the withdrawn number
	Domain 3	Unclear risk	No blinding was reported
	Domain 4	Unclear risk	No information given
	Domain 5	Low risk	No missing outcome data
	Domain 6	Low risk	All outcome data reported
	Domain 7	Low risk	No other bias detected
Konjen et al. [23]	Domain 1	Low risk	Block randomisation method using a computerised random number generator
	Domain 2	Low risk	Used sealed opaque envelopes with randomly assigned numbers
	Domain 3	Unclear risk	No blinding was reported
	Domain 4	Unclear risk	No information given
	Domain 5	Low risk	Noted all participants leaving or not completing the study
	Domain 6	Low risk	All outcome data reported
	Domain 7	Low risk	No other bias detected
Ulusoy et al. [24]	Domain 1	Low risk	One author used the stratified block randomisation method
	Domain 2	High risk	Allocated patients according to gender and BMI
	Domain 3	High risk	For practical reasons, no blinding was performed for the allocated treatment
	Domain 4	Unclear risk	No information given
	Domain 5	Low risk	Noted all participants leaving or not completing the study
	Domain 6	Low risk	All outcome data reported
	Domain 7	Low risk	No other bias detected
Akınoğlu et al. [25]	Domain 1	Unclear risk	No information given
	Domain 2	Low risk	Sealed envelope method was used for randomisation
	Domain 3	Low risk	Single-blinded
	Domain 4	Unclear risk	No information given
	Domain 5	Low risk	Noted all participants leaving or not completing the study
	Domain 6	Low risk	All outcome data reported
	Domain 7	High risk	Only female participants were included
Akınoğlu and Köse [26]	Domain 1	Unclear risk	No information given
	Domain 2	Low risk	Sealed envelope method was used for randomisation
	Domain 3	Low risk	Single-blinded
	Domain 4	Unclear risk	No information given
	Domain 5	Low risk	Noted all participants leaving or not completing the study
	Domain 6	Low risk	All outcome data reported
	Domain 7	High risk	Only female participants were included

**Table 3:** Newcastle-Ottawa scale to assess the quality of non-randomised studies.

Study	Selection	Comparability	Exposure
Cheing et al. [21]	****	*	***
Dedes et al. [27]	****	*	***

Discussion

ESWT showed a superior effect when compared with UST in terms of functional impairment, AOFAS scale score, and morning pain shown by the results of the analyses. Functional impairment (P = 0.22) and AOFAS scale score (P = 0.20) showed enhancements in the ESWT group compared with the UST group but failed to reach statistical significance (Figures 4, 5). Although pain during the first steps in the morning showed a trend favouring the ESWT group, statistical significance was not reached (P = 0.39) (Figure 2). Conversely, there was a statistically significant (P = 0.005) improvement in the analysis of the pain during activity for the ESWT group (Figure 3). In terms of between-study heterogeneity, it was low for AOFAS scale score (I2 = 0%), moderate for activity pain (I2 = 73%), and high for both morning pain (I2 = 100%) and functional impairment (I2 = 99%), according to the heterogeneity assessment mentioned in the methodological section.

Considering the secondary outcomes, ESWT group showed a significant improvement in primary efficacy success rate (P = 0.012) and activity limitations (P < 0.05) when compared to UST [24,25,27]. However, in terms of pain intensity after treatment, results varied amongst included studies [21,22,25-27]. With respect to the reduction of plantar fascia thickness, no statistically significant difference was found between the two groups [24].

There have been multiple studies in the literature about the best choice of treatment for plantar fasciitis. A study by Krishnan et al. showed additional positive reports that substantiate the effectiveness of ESWT on the treatment of plantar fasciitis by reporting the mean VAS scores to be decreased from an average of 9.2 to 3.4, at four weeks after treatment [30]. Additionally, a similar RCT found that ESWT had higher pain reduction compared to UST at three, six, and 12 weeks after the treatment [31]. The aforementioned studies support the current study’s findings, which can be attributed to the two proposed mechanisms of ESWT, namely, the inhibition of painful stimuli resulting in pain relief or the activation of the healing cascade [15,16]. Considering the data from the available studies, ESWT should be preferred over UST in the management of chronic plantar fasciitis.

A summary of the available evidence was provided in this review using a systematic approach as well as an assessment of the risk of bias of relevant studies and trials [21-24]. Five RCTs and two cohort studies were homogenous, based on the included population of interest and design. Therefore, this allows for a non-biased comparison. The combination of these factors makes the conclusions of the current study robust from the best available evidence. Nevertheless, the data of this paper should be studied in terms of inherent limitations. The identification of seven studies with a sample of 369 patients may not be sufficient to make definitive conclusions. Additional clinical trials with larger sample sizes are required to further evaluate the current findings.

## Conclusions

Although the evidence is limited with only seven studies with a total of 369 patients comparing the use of ESWT and UST, the results of this meta-analysis suggest that ESWT is a superior option in the treatment of patients with plantar fasciitis. This is because it improves the pain activity and intensity, primary efficacy success rate, activity limitations, and patient satisfaction. Additionally, ESWT does not worsen morning pain, functional impairment, AOFAS scale score, and the plantar fascial thickness compared to UST. The authors suggest that more clinical studies are required to further evaluate the current conclusions as well as the effectiveness of ESWT.

## References

[1] Rompe JD, Furia J, Weil L, Maffulli N (2007). Shock wave therapy for chronic plantar fasciopathy. Br Med Bull.

[2] Schwartz EN, Su J (2014). Plantar fasciitis: a concise review. Perm J.

[3] Thomas JL, Christensen JC, Kravitz SR (2010). The diagnosis and treatment of heel pain: a clinical practice guideline-revision 2010. J Foot Ankle Surg.

[4] Roxas M (2005). Plantar fasciitis: diagnosis and therapeutic considerations. Altern Med Rev.

[5] Singh D, Angel J, Bentley G, Trevino SG (1997). Fortnightly review. Plantar fasciitis. BMJ.

[6] Riddle DL, Pulisic M, Pidcoe P, Johnson RE (2003). Risk factors for plantar fasciitis: a matched case-control study. J Bone Joint Surg Am.

[7] Sahin N, Oztürk A, Atıcı T (2010). Foot mobility and plantar fascia elasticity in patients with plantar fasciitis. Acta Orthop Traumatol Turc.

[8] Lawrence DA, Rolen MF, Morshed KA, Moukaddam H (2013). MRI of heel pain. AJR Am J Roentgenol.

[9] Draghi F, Gitto S, Bortolotto C, Draghi AG, Ori Belometti G (2017). Imaging of plantar fascia disorders: findings on plain radiography, ultrasound and magnetic resonance imaging. Insights Imaging.

[10] Goff JD, Crawford R (2011). Diagnosis and treatment of plantar fasciitis. Am Fam Physician.

[11] Kiritsi O, Tsitas K, Malliaropoulos N, Mikroulis G (2010). Ultrasonographic evaluation of plantar fasciitis after low-level laser therapy: results of a double-blind, randomized, placebo-controlled trial. Lasers Med Sci.

[12] Schleberger R, Senge T (1992). Non-invasive treatment of long-bone pseudarthrosis by shock waves (ESWL). Arch Orthop Trauma Surg.

[13] Valchanou VD, Michailov P (1991). High energy shock waves in the treatment of delayed and nonunion of fractures. Int Orthop.

[14] Ogden JA, Alvarez R, Levitt R, Cross GL, Marlow M (2001). Shock wave therapy for chronic proximal plantar fasciitis. Clin Orthop Relat Res.

[15] Wang CJ, Huang HY, Pai CH (2002). Shock wave-enhanced neovascularization at the tendon-bone junction: an experiment in dogs. J Foot Ankle Surg.

[16] Rompe JD, Kirkpatrick CJ, Küllmer K, Schwitalle M, Krischek O (1998). Dose-related effects of shock waves on rabbit tendo Achillis. A sonographic and histological study. J Bone Joint Surg Br.

[17] Hooper PD (1996). Physical Modalities: A Primer for Chiropractic. https://www.google.co.in/books/edition/Physical_Modalities/6wFtAAAAMAAJ?hl=en.

[18] Kitchen SS, Partridge CJ (1990). A review of therapeutic ultrasound. Physiotherapy.

[19] Roebroeck ME, Dekker J, Oostendorp RA (1998). The use of therapeutic ultrasound by physical therapists in Dutch primary health care. Phys Ther.

[20] Lindsay DM, Dearness J, McGinley CC (1995). Electrotherapy usage trends in private physiotherapy practice in Alberta. Physiother Can.

[21] Cheing GLY, Chang H, Lo SK (2007). A comparison of the effectiveness of extracorporeal shock wave and ultrasound therapy in the management of heel pain. Shock Waves.

[22] Greve JM, Grecco MV, Santos-Silva PR (2009). Comparison of radial shockwaves and conventional physiotherapy for treating plantar fasciitis. Clinics (Sao Paulo).

[23] Konjen N, Napnark T, Janchai S (2015). A comparison of the effectiveness of radial extracorporeal shock wave therapy and ultrasound therapy in the treatment of chronic plantar fasciitis: a randomized controlled trial. J Med Assoc Thai.

[24] Ulusoy A, Cerrahoglu L, Orguc S (2017). Magnetic resonance imaging and clinical outcomes of laser therapy, ultrasound therapy, and extracorporeal shock wave therapy for treatment of plantar fasciitis: a randomized controlled trial. J Foot Ankle Surg.

[25] Akinoglu B, Köse N, Kirdi N, Yakut Y (2017). Comparison of the acute effect of radial shock wave therapy and ultrasound therapy in the treatment of plantar fasciitis: a randomized controlled study. Pain Med.

[26] Akınoğlu B, Köse N (2018). A comparison of the acute effects of radial extracorporeal shockwave therapy, ultrasound therapy, and exercise therapy in plantar fasciitis. J Exerc Rehabil.

[27] Dedes V, Tzirogiannis K, Polikandrioti M, Dede AM, Nikolaidis C, Mitseas A, Panoutsopoulos GI (2019). Radial extra corporeal shockwave therapy versus ultrasound therapy in the treatment of plantar fasciitis. Acta Inform Med.

[28] Moher D, Liberati A, Tetzlaff J, Altman DG (2009). Preferred reporting items for systematic reviews and meta-analyses: the PRISMA statement. Ann Intern Med.

[29] Wells GA, Shea B, O'Connell D (2000). The Newcastle-Ottawa Scale (NOS) for assessing the quality of nonrandomised studies in meta-analyses. http://www.ohri.ca/programs/clinical_epidemiology/oxford.asp.

[30] Krishnan A, Sharma Y, Singh S (2012). Evaluation of therapeutic effects of extracorporeal shock wave therapy in resistant plantar fasciitis patients in a tertiary care setting. Med J Armed Forces India.

[31] Kaewpinthong U, Hemtasilpa S, Phiphobmongkol U (2004). A comparison of the effects of low energy shock wave therapy and ultrasound for the treatment of plantar fasciitis. J Thai Rehabil Med.

